# Understanding *Pseudomonas* status among adults with cystic fibrosis: a real-world comparison of the Leeds criteria against clinicians’ decision

**DOI:** 10.1007/s10096-017-3168-4

**Published:** 2018-01-06

**Authors:** Zhe Hui Hoo, Frank Peter Edenborough, Rachael Curley, Laura Prtak, Jane Dewar, Mark Ivan Allenby, Julia Anne Nightingale, Martin James Wildman

**Affiliations:** 1 School of Health and Related Research (ScHARR), University of Sheffield, Regent Court, 30 Regent Street, Sheffield, S1 4DA UK; 2 Sheffield Adult Cystic Fibrosis Centre, Brearley Outpatient, Northern General Hospital, Herries Road, S5 7AU Sheffield, UK; 3 Wolfson Cystic Fibrosis Centre, City Hospital Campus NUH, Hucknall Road, Nottingham, NG5 1PB UK; 4 Department of Adult Cystic Fibrosis, University Hospital Southampton NHS Trust, Southampton General Hospital, Tremona Road, Southampton, SO16 6YD UK

## Abstract

*Pseudomonas aeruginosa* status influences cystic fibrosis (CF) clinical management but no ‘gold standard’ definition exists. The Leeds criteria are commonly used but may lack sensitivity for chronic *P. aeruginosa*. We compared clinicians’ decision with the Leeds criteria in three adult CF centres. Two independent prospective datasets (Sheffield dataset, *n* = 185 adults; ACtiF pilot dataset, *n* = 62 adults from two different centres) were analysed. Clinicians involved in deciding *P. aeruginosa* status were blinded to the study objectives. Clinicians considered more adults with CF to have chronic *P. aeruginosa* infection compared to the Leeds criteria. This was more so for the Sheffield dataset (106/185, 57.3% with clinicians’ decision vs. 80/185, 43.2% with the Leeds criteria; kappa coefficient between these two methods 0.72) compared to the ACtiF pilot dataset (34/62, 54.8% with clinicians’ decision vs. 30/62, 48.4% with the Leeds criteria; kappa coefficient between these two methods 0.82). However, clinicians across different centres were relatively consistent once age and severity of lung disease, as indicated by the type of respiratory samples provided, were taken into account. Agreement in *P. aeruginosa* status was similar for both datasets among adults who predominantly provided sputum samples (kappa coefficient 0.78) or adults > 25 years old (kappa coefficient 0.82). Across three different centres, clinicians did not always agree with the Leeds criteria and tended to consider the Leeds criteria to lack sensitivity. Where disagreement occurred, clinicians tended to diagnose chronic *P. aeruginosa* infection because other relevant information was considered. These results suggest that a better definition for chronic *P. aeruginosa* might be developed by using consensus methods to move beyond a definition wholly dependent on standard microbiological results.

## Introduction

Cystic fibrosis (CF), an autosomal recessive genetic condition, affects around 10,000 people in the UK [1]. It is a multi-system condition; lungs (resulting in recurrent infections and respiratory failure) and the gastrointestinal tract (resulting in malabsorption of fat and poor growth) are the two main affected organs.

*Pseudomonas aeruginosa* remains the most common chronic lung pathogen among adults with CF, despite changes in pathogen epidemiology [1, 2]. Once acquired, *P. aeruginosa* infection is associated with increased risk of pulmonary exacerbations and accelerated lung function decline [3]. Early eradication therapy mitigates lung function decline and delays onset of chronic infection [4], while long-term inhaled antibiotics reduce the risk of exacerbation and improves lung function in those with chronic *P. aeruginosa* infection [5]. *Pseudomonas aeruginosa* status influences various clinical decisions in CF [5–7]. During clinic reviews and in-patient stays, adults with CF not infected by *P. aeruginosa* are segregated from those with *P. aeruginosa* infection to prevent cross-infection [7]. Adults with CF deemed to have chronic *P. aeruginosa* infection should be treated with long-term inhaled anti-pseudomonal antibiotics [5], because chronic *P. aeruginosa* infection is associated with accelerated lung function decline. They should also be treated with appropriately broad spectrum intravenous antibiotics during pulmonary exacerbations [7, 8], due to the resistance pattern of *P. aeruginosa*. Careful monitoring of *P. aeruginosa* status is, hence, an important standard of care in CF [8].

However, there is no ‘gold standard’ to define *P. aeruginosa* status among adults with CF. One of the most commonly used definitions in CF research are the Leeds criteria [7]. These criteria define “chronic *P. aeruginosa* infection” as > 50% of months with cough swabs/sputum cultures in the preceding 12 months that were positive for *P. aeruginosa* [7]. For example, if an adult provided nine sputum samples over seven months in the last year and *P. aeruginosa* was cultured in four of those months, that would be considered as chronic *P. aeruginosa*. “Intermittent *P. aeruginosa* infection” was defined as ≤ 50% of months with positive *P. aeruginosa* culture. In the above example, if only three of those months have at least one positive *P. aeruginosa* culture, that would be considered as intermittent *P. aeruginosa*, even if majority of the samples (e.g. five of nine) were positive for *P. aeruginosa*. Therein lies the advantage of the Leeds criteria over other definitions, e.g. Ballmann et al.’s definition of chronic *P. aeruginosa* as > 50% of cough swabs/sputum cultures being *P. aeruginosa* positive in a 12-month period [9]. If there is a new positive culture for *P. aeruginosa*, more samples might be taken within a short interval, especially if there was admission for intravenous antibiotics as part of the eradication regime. More intensive monitoring could bias towards misclassifying intermittent *P. aeruginosa* as chronic if the timings of samples taken were not considered.

Whilst the Leeds criteria are very specific for chronic *P. aeruginosa* infection, recent studies using polymerase chain reaction (PCR) techniques have shown that the Leeds criteria are insensitive, with a tendency to under-diagnose chronic *P. aeruginosa* as intermittent infection [10, 11]. A particular issue is the equal weighting given to cough swabs and sputum samples, even though a cough swab is less sensitive in culturing *P. aeruginosa* [12, 13]. In addition, the Leeds criteria do not consider other key information, such as *P. aeruginosa* strain typing or results of other investigations, e.g. serum *P. aeruginosa* antibody levels [14]. For example, resistant epidemic strains are less likely to be cleared than unique environmental strains [15]. In their day-to-day work, it is likely that clinicians assimilate all of this relevant information in making a decision about *P. aeruginosa* status.

However, how clinicians reach a decision on *P. aeruginosa* status has not been formally investigated. It is also important to understand how different approaches to the definition of *P. aeruginosa* status will affect reported prevalence within routine datasets, such as the UK CF Registry. Using two independent prospective datasets, we set out to compare the distribution of *P. aeruginosa* status as judged by clinicians with the distribution defined by the Leeds criteria. We also systematically compared clinicians’ decisions against the Leeds criteria to identify subgroups of adults where clinicians are more likely to disagree with the Leeds criteria.

## Materials and methods

### Design and setting

This is a cross-sectional analysis of prospectively collected data from two independent datasets and includes clinicians from three adult CF centres.

The first dataset included every eligible adult receiving care at the Sheffield Adult Cystic Fibrosis Centre in 2015 (*n* = 185). Regulatory approval to analyse data from this dataset was obtained from NHS Health Research Authority (IRAS number 210313).

The second dataset included every eligible participant in the ACtiF pilot study. Participants were recruited from the East Midlands Wolfson Cystic Fibrosis Centre (*n* = 31) and the Wessex Adult Cystic Fibrosis Centre (*n* = 31). This is a randomised controlled external pilot trial to evaluate the feasibility for a full-scale randomized controlled trial (RCT) of a theory-based complex intervention to support self-care and adherence with inhaled therapies (ISRCTN13076797). Regulatory approval for the pilot was obtained from Brent Research Ethics Committee (REC reference 16/LO/0356).

Patients with lung transplantation or who were on ivacaftor were excluded. Lung transplantation changes lung microbiome, which complicates the interpretation of *P. aeruginosa* status [16]. Ivacaftor reduces the likelihood of culturing *P. aeruginosa*, which may affect the diagnostic properties of the Leeds criteria [17].

### Data collection

For the Sheffield dataset, two experienced CF physicians (FPE, RC) independently reviewed all relevant patient data up to December 2015 to decide on the *P. aeruginosa* status (‘no *P. aeruginosa*’, ‘intermittent’, ‘chronic’). If agreement was achieved at this stage, it was accepted as the clinicians’ status. If agreement was not achieved, the *P. aeruginosa* status was decided in a consensus meeting by both physicians and a microbiologist with CF experience (LP), whereby all three clinicians agreed on the final clinicians’ status. The following data for 2015 were obtained from electronic patient records:

- Microbiology: every cough swab and sputum sample in 2015
- Demographics: age, gender, pancreatic status, presence of CF-related diabetes, socioeconomic status (postcodes were used to derive the Index of Multiple Deprivation, IMD [18])
- Body mass index, BMI (in kg/m^2^)
- %FEV_1_ (calculated with the Knudson equation [19])
- Annual intravenous antibiotic days (in number of days)

For the ACtiF pilot dataset, an experienced CF physician at each centre (JD at the East Midlands Wolfson Cystic Fibrosis Centre; MIA or JAN at the Wessex Adult Cystic Fibrosis Centre) reviewed all relevant patient data up to the point of participant recruitment to decide on *P. aeruginosa* status. A similar set of data to the Sheffield dataset was collected at the point of recruitment from June to September 2016. Participants’ %FEV_1_ at baseline was calculated using the Global Lung Function Initiative (GLI) equation [20].

All clinicians involved in deciding the *P. aeruginosa* status were blinded to the study objectives and analyses plan to minimise bias. Across all sites, microbiology samples were collected during every clinical review, and care standards dictate that the interval between reviews should be ≤ 3 months. *Pseudomonas aeruginosa* was isolated from samples in the respective nationally accredited microbiology laboratories in Sheffield, Nottingham, Southampton and Poole using standard methods in accordance to national guidelines [21].

### Data analysis

In this analysis, the Leeds criteria categories of ‘free from infection’ and ‘never’ were combined into a single category (‘no *P. aeruginosa*’) because both these categories are typically treated in the same manner among adults with CF. This produced three categories of *P. aeruginosa* status, i.e. ‘no *P. aeruginosa*’ if no growth of *P. aeruginosa* in cough swabs/sputum cultures during the previous 12 months; ‘intermittent’ if *P. aeruginosa* positive in ≤ 50% of months when cough swabs/sputum cultures had been taken; and ‘chronic’ if *P. aeruginosa* positive in > 50% of months when cough swabs/sputum cultures had been taken.

Data from the Sheffield and ACtiF pilot datasets were analysed separately. Reasons for every clinicians’ decision in the Sheffield dataset were recorded and thematically analysed to understand the basis of clinicians’ decision-making.

For both datasets, appropriate descriptive statistics were generated, including cross-tabulation of the Leeds criteria *P. aeruginosa* categories against clinicians’ decision. Agreement between clinicians’ decision and the Leeds criteria were determined using kappa statistics [22]. Sensitivity, specificity and likelihood ratios of the Leeds criteria in diagnosing chronic *P. aeruginosa* were calculated using clinicians’ decision as the reference. Likelihood ratios were calculated instead of predictive values because likelihood ratios are independent of prevalence, hence comparable between the two datasets [23].

Clinical characteristics for the group in which the *P. aeruginosa* status differed between clinicians’ decision and the Leeds criteria were compared using non-parametric methods against the group where there was agreement, to identify the subgroups of adults whereby the *P. aeruginosa* status is more contentious.

All analyses were performed using SPSS Statistics v22 (IBM Corp.), with *p*-values < 0.05 considered statistically significant.

## Results

For the Sheffield dataset, 185 adults were included, with median age of 27 years [interquartile range (IQR) 20–34 years]. A total of 1211 relevant respiratory samples were collected during 2015 (750 sputum samples, 461 cough swabs). For the ACtiF pilot dataset, 62 adults were included, with median age of 28 years (IQR 22–37 years). A total of 528 relevant respiratory samples were collected in a one-year period prior to recruitment (491 sputum samples, 37 cough swabs). The ACtiF pilot participants have more severe lung disease, even after allowing for the difference in %FEV_1_ measurements, which is, in part, due to the eligibility criteria of the ACtiF pilot. To be eligible, participants must be on long-term inhaled therapies and this selected for adults with more severe lung disease. Not surprisingly, the average number of respiratory samples collected from the ACtiF pilot was higher and predominantly sputum, whereas the Sheffield study subjects with less severe lung disease produced far more cough swabs (see Table 1).

**Table 1:** Demographic and clinical characteristics of the study subjects

	Sheffield dataset^a^ (*n* = 185)	ACtiF pilot dataset (*n* = 62)
Age in years, median (IQR)	27 (20–34)	28 (22–37)
Females (%)	87 (47.0)	26 (41.9)
Pancreatic insufficient (%)	142 (76.8)	54 (87.1)
CF-related diabetes (%)	42 (22.7)	28 (45.2)
Social deprivation (IMD quintile)		
1 (least deprived) (%)	28 (15.1)	15 (24.2)
2 (%)	16 (8.6)	14 (22.6)
3 (%)	43 (23.2)	15 (24.2)
4 (%)	44 (23.8)	11 (17.7)
5 (most deprived) (%)	54 (29.3)	7 (11.3)
Number of relevant microbiological samples		
Cough swabs, median (IQR)	2 (0–4)	0 (0–0)
Sputum samples, median (IQR)	3 (1–7)	8 (5–10)
Total, median (IQR)	6 (4–9)	8 (6–10)
*Pseudomonas aeruginosa* status (Leeds criteria)		
Chronic *P. aeruginosa* (%)	80 (43.2)	30 (48.4)
Intermittent *P. aeruginosa* (%)	31 (16.8)	8 (12.9)
No *P. aeruginosa* (%)	74 (40.0)	24 (38.7)
*Pseudomonas aeruginosa* status (clinicians’ decision)		
Chronic *P. aeruginosa* (%)	106 (57.3)	34 (54.8)
Intermittent *P. aeruginosa* (%)	15 (8.1)	6 (9.7)
No *P. aeruginosa* (%)	64 (34.6)	22 (35.5)
BMI in kg/m^2^, median (IQR)	23.0 (20.3–26.0)	22.2 (19.6–25.4)
% predicted FEV_1_, median (IQR)	81 (63–93)^b^	51.2 (42.9–77.9)^c^
Annual intravenous antibiotic days, median (IQR)	14 (0–35)	17 (5–44)

^a^Complete data were available for every clinical variable in the Sheffield dataset, except for one study subject who did not have any FEV_1_ readings in 2015 due to inability to perform spirometry testing. Complete data were available for every clinical variable in the ACtiF pilot dataset

^b^This is the highest FEV_1_ obtained in 2015. % predicted is calculated using the Knudson equation

^c^This is the baseline FEV_1_ at recruitment to the ACtiF pilot. % predicted is calculated using the GLI equation

In both datasets, more adults have chronic *P. aeruginosa* infection according to clinicians’ decision compared to the Leeds criteria. This was more so for the Sheffield dataset (106/185, 57.3% with clinicians’ decision vs. 80/185, 43.2% with the Leeds criteria) compared to the ACtiF pilot dataset (34/62, 54.8% with clinicians’ decision vs. 30/62, 48.4% with the Leeds criteria). Where there was disagreement in *P. aeruginosa* status, the clinicians tended to diagnose chronic *P. aeruginosa* infection, but the Leeds criteria diagnosed no or intermittent *P. aeruginosa* (see Tables 2 and 3). For example, 21 adults in the Sheffield dataset fulfilled the Leeds criteria for ‘intermittent *P. aeruginosa*’ but were deemed by clinicians to have ‘chronic *P. aeruginosa*’. This disagreement was, in part, driven by clinicians placing different weighting on cough swabs compared to sputum samples, and clinicians diagnosed chronic *P. aeruginosa* infection even if *P. aeruginosa* was less frequently cultured on cough swabs, i.e. cough swabs with no growths tended to be ignored (see the reasons provided by Sheffield clinicians for diagnosing chronic *P. aeruginosa* in Table 4).

**Table 2:** *Pseudomonas aeruginosa* status according to clinicians’ decision vs. the Leeds criteria

Leeds criteria *P. aeruginosa* status, number of adults	Sheffield dataset^a^ (*n* = 185) *P. aeruginosa* status according to clinicians’ decision, number of adults			ACtiF pilot dataset^b^ (*n* = 62) *P. aeruginosa* status according to clinicians’ decision, number of adults		
	No *P. aeruginosa*	Intermittent *P. aeruginosa*	Chronic *P. aeruginosa*	No *P. aeruginosa*	Intermittent *P. aeruginosa*	Chronic *P. aeruginosa*
No *P. aeruginosa*	64	5	5	21	1	2
Intermittent *P. aeruginosa*	0	10	21	1	4	3
Chronic *P. aeruginosa*	0	0	80	0	1	29

^a^Cohen’s kappa coefficient of 0.72 [95% confidence interval (CI) 0.64–0.80] between clinicians’ decision and the Leeds criteria for the Sheffield dataset

^b^Cohen’s kappa coefficient of 0.82 (95% CI 0.74–0.89) between clinicians’ decision and the Leeds criteria for the ACtiF pilot dataset

**Table 3:** Diagnostic properties of the Leeds criteria for ‘chronic *P. aeruginosa* infection’, in comparison to clinicians’ decision

	Sheffield dataset (*n* = 185)	ACtiF pilot dataset (*n* = 62)
Sensitivity (95% CI)	0.75 (0.66–0.83)	0.85 (0.69–0.95)
Specificity (95% CI)	1.00 (0.94–1.00)	0.96 (0.82–1.00)
Positive likelihood ratio (95% CI)	Infinity	23.88 (3.47–164.49)
Negative likelihood ratio (95% CI)	0.25 (0.18–0.34)	0.15 (0.07–0.34)

**Table 4:** Reasons provided by Sheffield clinicians for deciding when an adult with cystic fibrosis (CF) has chronic *P. aeruginosa* infection

Clinicians’ basis for chronic *P. aeruginosa* infection
Multiple positive samples (≥ 3 months with positive samples in a year)
If cough swab only, at least two positive cough swabs in a year
Accept as chronic *P. aeruginosa* infection even with negative samples, if poor quality respiratory sample^a^ and high serum *P. aeruginosa* antibody levels
Accept as chronic *P. aeruginosa* infection even with negative samples, if high adherence to inhaled antibiotics
Accept as chronic *P. aeruginosa* infection even with negative samples, if clinically deteriorates with cessation of inhaled antibiotics
Transmissible strain difficult to eradicate, so accept as chronic *P. aeruginosa* infection if cultured within the last 12–18 months
Mucoid *Pseudomonas* difficult to eradicate, so accept as chronic *P. aeruginosa* infection if cultured within the last 12–18 months
Two separate positive cultures of *P. aeruginosa* > 1 year apart of the same strain
Long previous history of *P. aeruginosa* infection (first positive culture > 5 years ago), with ≥ 2 positive *P. aeruginosa* cultures > 1 year apart

^a^A particular respiratory sample (cough swab/sputum sample) was deemed as possibly ‘poor quality’ if no other known CF pathogens, e.g. *Haemophilus influenzae*, was cultured from that sample

In the Sheffield dataset, the most obvious differences between adults with discordant and concordant *P. aeruginosa* status were the numbers of cough swabs and sputum samples. The discordant group were also younger (see Table 5). This would account for the minor differences in BMI and %FEV_1_ between these two groups. The smaller sample size and more homogenous nature of the ACtiF pilot dataset made it more difficult to detect differences between adults with discordant versus concordant *P. aeruginosa* status. Nonetheless, the differences in age and %FEV_1_ were in the same direction as the Sheffield dataset, suggesting some consistency in the decision-making by different clinicians across three CF centres. These differences allowed the identification of two broad subgroups in which clinicians were more likely to disagree with the Leeds criteria: (1) those who provided more cough swabs than sputum samples and (2) younger adults (≤ 25 years).

**Table 5:** Demographic and clinical characteristics for study subjects with discordant vs. concordant *P. aeruginosa* status between clinicians’ decision and the Leeds criteria

	Sheffield dataset			ACtiF pilot dataset		
	Discordant *P. aeruginosa* status (*n* = 31)	Concordant *P. aeruginosa* status (*n* = 154)	*p*-Value	Discordant *P. aeruginosa* status (*n* = 8)	Concordant *P. aeruginosa* status (*n* = 54)	*p*-Value
Age in years, median (IQR)	20 (18–26)	27 (22–34)	0.001*	25 (22–31)	28 (22–38)	0.266*
Females (%)	15 (48.4)	72 (46.8)	0.868***	3 (37.5)	23 (42.6)	1.000****
Pancreatic insufficient (%)	30 (96.8)	112 (72.7)	0.004***	8 (100.0)	46 (85.2)	0.581****
CF-related diabetes (%)	5 (16.1)	37 (24.0)	0.338***	3 (37.5)	25 (46.3)	0.719****
Social deprivation (IMD quintile)						
1 (least deprived) (%)	7 (22.6)	22 (14.3)	0.265**	1 (12.5)	14 (25.9)	0.875**
2 (%)	1 (3.1)	15 (9.7)		2 (25.0)	12 (22.2)	
3 (%)	10 (32.3)	33 (21.4)		4 (50.0)	11 (20.4)	
4 (%)	6 (19.4)	37 (24.1)		1 (25.0)	10 (18.5)	
5 (most deprived) (%)	7 (22.6)	47 (30.5)		0	7 (13.0)	
Number of microbiological samples						
Cough swabs, median (IQR)	4 (2–7)	1 (0–4)	< 0.001*	0 (0–2)	0 (0–0)	0.796*
Sputum samples, median (IQR)	1 (1–3)	4 (1–7)	0.002*	8 (3–10)	8 (5–10)	0.613*
Total, median (IQR)	6 (4–8)	6 (4–9)	0.987*	9 (5–10)	8 (6–10)	0.728*
Sputum producers^a^ (%)	21 (67.7)	118 (76.6)	0.297***	8 (100)	51 (94.4)	1.000****
BMI in kg/m^2^, median (IQR)	23.0 (21.3–26.0)	22.9 (20.1–30.3)	0.631*	20.8 (17.7–23.7)	22.2 (19.9–25.7)	0.163*
% predicted FEV_1_, median (IQR)	87 (75–94)^b^	80 (61–93)^b^	0.292*	55.6 (40.6–81.7)^c^	49.6 (42.9–76.2)^c^	0.600*
Annual intravenous antibiotic days, median (IQR)	12 (0–37)	14 (0–34)	0.460*	14 (4–35)	21 (5–45)	0.575*

^a^A person was considered a ‘sputum producer’ if he/she provided at least one sputum sample over a one-year period

^b^This is the highest FEV_1_ in 2015, with % predicted calculated using the Knudson equation

^c^This is the baseline FEV_1_ at recruitment, with % predicted calculated using the GLI equation

*Mann–Whitney test; **Chi-squared test for trend; ***Chi-squared test; **** Fisher’s exact test

Most of the discrepancies between clinicians and the Leeds criteria in both datasets were driven by these ‘more difficult to agree’ subgroups (see Table 6). The extent by which clinicians diagnosed more chronic *P. aeruginosa* in relation to the Leeds criteria were actually very similar across both datasets among adults who provided at least an equal number of sputum samples as cough swabs, or older adults (i.e. the ‘easier to agree’ subgroups). Among adults > 25 years old who provided at least an equal number of sputum samples as cough swabs, the difference in the proportion of adults with chronic *P. aeruginosa* according to clinicians versus the Leeds criteria were similar for both datasets (Sheffield: 59/74, 79.7% with clinicians’ decision vs. 54/74, 73.0% with the Leeds criteria; ACtiF pilot: 22/36, 61.1% with clinicians’ decision vs. 20/36, 55.6% with the Leeds criteria).

**Table 6:** Comparison of the *P. aeruginosa* status according to the Leeds criteria vs. clinicians’ decision, stratified according to different subgroups

	Sheffield dataset (*n* = 185)			ACtiF pilot dataset (*n* = 62)		
	Number of adults in each subgroup (%)	Agreement between the Leeds criteria and clinicians’ decision, Cohen’s kappa coefficient (95% CI)	‘Sensitivity’ of the Leeds criteria in diagnosing chronic *P. aeruginosa* compared to clinicians’ decision (95% CI)	Number of adults in each subgroup (%)	Agreement between the Leeds criteria and clinicians’ decision, Cohen’s kappa coefficient (95% CI)	‘Sensitivity’ of the Leeds criteria in diagnosing chronic *P. aeruginosa* compared to clinicians’ decision (95% CI)
Overall	185	0.72 (0.64–0.80)	0.75 (0.66–0.83)	62	0.82 (0.74–0.89)	0.85 (0.69–0.95)
Subgroups based on microbiology samples						
Cough swabs > sputum samples (the ‘more difficult to agree’ subgroup)	77 (41.6)	0.54 (0.40–0.69)	0.38 (0.20–0.59)	5 (8.1)	N/A (all 5 adults were ‘no *P. aeruginosa*’ according to the Leeds criteria)	N/A (all 5 adults were non-chronic *P. aeruginosa*)
Sputum samples ≥ cough swabs (the ‘easier to agree’ subgroup)	108 (58.4)	0.78 (0.67–0.90)	0.88 (0.78–0.94)	57 (91.9)	0.78 (0.64–0.93)	0.85 (0.69–0.95)
Subgroups based on age						
Age ≤ 25 years (the ‘more difficult to agree’ subgroup)	85 (45.9)	0.60 (0.47–0.72)	0.53 (0.34–0.69)	24 (38.7)	0.72 (0.48–0.95)	0.75 (0.43–0.95)
Age > 25 years (the ‘easier to agree’ subgroup)	100 (54.1)	0.82 (0.72–0.93)	0.88 (0.78–0.95)	38 (61.3)	0.82 (0.65–0.98)	0.91 (0.71–0.99)

## Discussion

Although *P. aeruginosa* status and management decisions related to *P. aeruginosa* status are often decided by clinicians in routine clinical practice, this is the first study that formally evaluates clinicians’ diagnosis of *P. aeruginosa* status among adults with CF. This study demonstrated that clinicians diagnosed chronic *P. aeruginosa* when the Leeds criteria did not, partly because clinicians placed less importance on negative cough swabs, and, in the face of negative cough swabs, integrated other relevant information to decide on *P. aeruginosa* status. On the other hand, the Leeds criteria depend solely on standard microbiological results and ignore other key information, such as *P. aeruginosa* strain typing.

This finding is consistent with previous studies which found cough swabs less sensitive in culturing *P. aeruginosa* [12, 13] and the Leeds criteria lacking in sensitivity for chronic *P. aeruginosa* when compared against PCR methods [10, 11]. Indeed, quantitative PCR assays often detect *P. aeruginosa* when bacterial cultures are negative [24]; hence, a negative culture does not always imply the absence of *P. aeruginosa* infection and intermittent positive *P. aeruginosa* cultures do not always imply intermittent infection.

It may appear that Sheffield clinicians diagnosed far more chronic *P. aeruginosa* cases versus the Leeds criteria in comparison to the ACtiF pilot clinicians. However, the observed discrepancy was largely driven by the differences in the case mix (age and severity of lung disease, as indicated by the type of respiratory samples provided) between the two datasets. Among the subgroups in which agreement between clinicians’ decision and the Leeds criteria were more likely (i.e. older adults or adults who provided at least equal numbers of sputum samples to cough swabs), the discrepancies between the Leeds criteria and clinicians’ decision for both datasets were very similar, suggesting a degree of consistent decision-making by clinicians from three different specialist adult CF centres in the UK. Clinicians involved in deciding *P. aeruginosa* status were blinded to the study objectives and analyses plan to minimise bias. The results from three centres were compared in this study, helping us to gain insight into patterns across adult UK centres rather than the peculiarities specific to a single centre.

Limitations of this study should be acknowledged. The limited number of study subjects with ‘discordant *P. aeruginosa* status’ meant that multivariate logistic regression could not be used to analyse differences in clinical characteristics between the discordant and concordant groups. Data on whether respiratory samples were collected during periods on or off anti-pseudomonal antibiotics were not collected, and, therefore, we could not explore whether this is a specific factor that causes discordance between clinicians’ decision and the Leeds criteria. *Pseudomonas aeruginosa* was isolated from respiratory samples using standard *Pseudomonas* isolation agar, as recommended by national guidelines [21], but more sensitive culture methods, e.g. quantitative cultures, may well increase the proportion of adults diagnosed with chronic *P. aeruginosa* infection according to the Leeds criteria. However, evaluation of novel culture methods that are not routinely available is beyond the scope of this pragmatic study. In the two ACtiF pilot centres, a single clinician decided on the *P. aeruginosa* status of participants recruited to the study after considering relevant data without a consensus process involving other clinicians, whereas Sheffield clinicians made decisions across the whole centre population with a relatively structured process involving independent consideration by two clinicians, followed by final consensus involving a third clinician. The Sheffield process allowed more detailed exploration of the challenge of determining *P. aeruginosa* status in the real world. For example, clinical deterioration after cessation of inhaled antibiotics despite negative samples. However, these ‘subjective’ reasons were only applied to a small minority of study subjects and such reasons were often triangulated with other ‘more objective’ data (e.g. high serum *P. aeruginosa* antibody levels) before a consensus ‘chronic *P. aeruginosa*’ decision was reached. Given the challenging nature of determining *P. aeruginosa* status, these data are valuable in making the case for further work to create an approach to improve diagnostic consistency. Clinicians across three different specialist adult CF centres displayed some consistency in their decision-making but they did not always agree with the Leeds criteria in diagnosing *P. aeruginosa* status, and the extent of this disagreement was influenced by the case mix.

The lack of a ‘gold standard’ for *P. aeruginosa* status means that different clinical trials evaluating treatments for people with chronic *P. aeruginosa* (e.g. nebulised levofloxacin and tobramycin inhalation powder) used different eligibility criteria [25, 26]. The exact definition of *P. aeruginosa* status is, perhaps, not crucial in a drug trial whereby people with uncertain *P. aeruginosa* status would be distributed randomly across different arms of the trial. However, an accurate *P. aeruginosa* status is crucial for a nebuliser adherence trial such as ACtiF because *P. aeruginosa* status influences the prescription of inhaled therapies and determines normative adherence (which is an outcome measure in the ACtiF pilot) [6, 27]. The challenge of determining *P. aeruginosa* status among adults with CF has clear parallels with the problem posed by exacerbation and identifies the need for a pragmatic set of guidelines that can be easily applied in clinical trials, in a similar way in which the Fuchs or EPIC criteria have been used to determine the presence of pulmonary exacerbations [28]. Both the Fuchs and EPIC criteria emerged out of the necessity to standardise the definition of exacerbations in multi-centre trials. Neither the Fuchs nor EPIC criteria would constitute the ‘gold standard’, but, nevertheless, provide an agreed definition that allows exacerbation to be diagnosed as a valid outcome in multi-centre clinical trials. Likewise, a standardised *P. aeruginosa* status definition will be important in a multi-centre nebuliser adherence trial such as the ACtiF study, which uses ‘normative adherence’ [6] as one of the secondary outcomes. Of note, both the Fuchs and EPIC criteria consist of ‘objective’ evidence, e.g. acute FEV_1_ decline, and more ‘subjective’ evidence, e.g. change in symptoms because exacerbations do not always present in the same way [28]. A similar approach might enable a consensus group to add additional parameters to the Leeds criteria.

Our findings may also have clinical implications. Preventative inhaled therapies are crucial to maintain health among people with CF [5], and the most potent of these are inhaled antibiotics. The decision to initiate inhaled antibiotics is predominantly driven by *P. aeruginosa* status. The results of this study suggest that *P. aeruginosa* status may be more difficult to define among younger adults who are particularly vulnerable to lung function decline [29]. Inadequate prescription of treatment (‘therapeutic inertia’) is the second biggest cause of treatment under-utilisation after low adherence [30]. It is crucial to ensure that a diagnosis of chronic *P. aeruginosa* is not inadvertently missed among younger adults who are likely to have accumulated less lung damage, have lower *P. aeruginosa* density in their lungs and reduced likelihood of culturing *P. aeruginosa* with standard respiratory samples. A consensus approach to the challenge of diagnosing chronic *P. aeruginosa* in this age group might prompt helpful approaches; for example, perhaps younger adults with CF should be monitored more intensively (e.g. supplementing standard respiratory samples with regular serum *P. aeruginosa* antibodies).

The next step of this research is to use formal consensus methods to integrate the expertise of clinicians from a greater number of adult CF centres in order to explicitly develop a pragmatic definition of chronic *P. aeruginosa* infection that moves beyond solely depending on standard microbiological results. Such a consensus exercise has been successfully completed, and a paper describing the results is in preparation.

Given that a perfectly sensitive and specific test for chronic *P. aeruginosa* is unlikely to become routinely available in the near future, we anticipate that a carefully developed set of consensus criteria to define chronic *P. aeruginosa* infection has the potential to contribute to clinical practice and bring similar benefits to those brought by the Fuchs criteria as a tool for structuring investigations around exacerbations.
